# Effects of local cryotherapy on systemic endothelial activation, dysfunction, and vascular inflammation in adjuvant-induced arthritis (AIA) rats

**DOI:** 10.1186/s13075-022-02774-1

**Published:** 2022-04-29

**Authors:** C. Peyronnel, P. Totoson, V. Petitcolin, F. Bonnefoy, X. Guillot, P. Saas, F. Verhoeven, H. Martin, C. Demougeot

**Affiliations:** 1 PEPITE EA4267, FHU INCREASE, Univ. Bourgogne Franche-Comté, F-25000 Besançon, France; 2 INSERM UMR 1098 RIGHT, EFS BFC, Univ. Bourgogne Franche-Comté, LabEX LipSTIC, F-25000 Besançon, France; 3 Service de Rhumatologie, CHU Felix Guyon, Ile de la Réunion, Saint-Denis, France; 4 Service de Rhumatologie, CHRU Besançon, F-25000 Besançon, France

**Keywords:** Arthritis, Local cryotherapy, Endothelial activation, Vascular inflammation, Endothelial dysfunction

## Abstract

**Aim:**

This study explored the systemic vascular effects of local cryotherapy with a focus on endothelial changes and arterial inflammation in the model of rat adjuvant-induced arthritis (AIA).

**Methods:**

Cryotherapy was applied twice a day on hind paws of AIA rats from the onset of arthritis to the acute inflammatory phase. Endothelial activation was studied in the aorta by measuring the mRNA levels of chemokines (CXCL-1, MCP-1 (CCL-2), MIP-1α (CCL-3)) and adhesion molecules (ICAM-1, VCAM-1) by qRT-PCR. Endothelial dysfunction was measured in isolated aortic and mesenteric rings. Aortic inflammation was evaluated *via* the mRNA expression of pro-inflammatory cytokines (TNF-α, IL-6) by qRT-PCR and leucocyte infiltration analysis (flow cytometry). Plasma levels of TNF-α, IL-6, IL-1β, IL-17A, and osteoprotegerin (OPG) were measured using Multiplex/ELISA.

**Results:**

AIA was associated with an increased aortic expression of CXCL-1 and ICAM-1 as well as an infiltration of leucocytes and increased mRNA expression of IL-6, IL-1β, and TNF-α. Local cryotherapy, which decreased arthritis score and structural damages, reduced aortic mRNA expression of CXCL-1, IL-6, IL-1β, and TNF-α, as well as aortic infiltration of leucocytes (T lymphocytes, monocytes/macrophages, neutrophils) and improved acetylcholine-induced vasorelaxation in the aorta and mesenteric arteries. Plasma levels of IL-17A and OPG were significantly reduced by cryotherapy, while the number of circulating leucocytes was not. IL-17A levels positively correlated with endothelial activation and dysfunction.

**Conclusion:**

In the AIA model, local cryotherapy reduced systemic endothelial activation, immune cell infiltration, and endothelial dysfunction. Mechanistically, the reduction of circulating levels of IL-17A appears as the possible link between joint cooling and the remote vascular effects.

**Supplementary Information:**

The online version contains supplementary material available at 10.1186/s13075-022-02774-1.

## Introduction

Rheumatoid arthritis (RA), the most common systemic inflammatory disorder which primarily involves synovial joints, is associated with higher cardiovascular (CV) mortality [1]. Despite the encouraging trend in the decline in CV diseases using an active treatment of RA, recent data from large population-based cohort studies indicated that CV morbidity remained higher compared with the general population [2, 3]. This excessive CV risk is only partly explained by traditional CV disease risk factors [1, 4]. Other factors, such as dysregulated immunity, iatrogenic, systemic inflammation, or yet unknown RA-related factors are likely involved [5]. Many of these factors affect CV health through changes in the endothelium. In a wide range of CV diseases, endothelial activation and dysfunction precede and initiate atherosclerosis, and their correction was associated with a reduced CV risk [6, 7]. Endothelial cell activation (EA) is defined by the activation of gene transcription in endothelial cells, leading to increased expression of adhesion molecules and the production of cytokines and chemokines. EA induces changes in endothelium permeability, favoring leucocyte recruitment and margination, and vascular inflammation. Endothelial dysfunction (ED) is a reversible alteration of the endothelial phenotype leading to an endothelium more prone to vasoconstriction. While ED has been largely documented in patients with early or established RA [8–10] as well as in animal models of arthritis [11], less data are available on EA or arterial wall inflammation.

In recent years, a renewed interest emerged in RA for cryotherapy as a well-tolerated and cost-effective adjunct therapy [12]. In patients with RA, local cryotherapy and whole body cryotherapy applied twice a day for 7 to 15 days significantly reduced pain and disease activity [12]. Using the adjuvant-induced arthritis (AIA) model in rats, the positive effect of local cryotherapy on arthritis severity and joint damage was shown to be IL-6/IL-17A-driven but TNF-α independent [13]. As a link between the decrease in disease activity, inflammation, and CV risk is highly suspected in *chronic inflammatory rheumatic* diseases [14], the hypothesis has emerged that local cryotherapy might extend its positive effects to the systemic vasculature.

The aim of the present study was to explore the systemic vascular effects of subchronic local cryotherapy in AIA rats, with a focus on endothelial changes and arterial inflammation. First, mRNA expression of EA markers such as adhesion molecules (ICAM-1, VCAM-1), cytokines/chemokines (CXCL-1, the murine equivalent of human IL-8 [15], MCP-1 (CCL-2), MIP-1α (CCL-3), TNF-α, IL-6), and arterial wall leucocyte infiltration was studied in aorta from AIA as compared to control rats. Second, the effect of local cryotherapy applied from the onset of arthritis to the maximal inflammatory phase on EA and arterial inflammation was measured. Third, whether the treatment reduced ED and enzymes whose overexpression is involved in ED (Arginase-2, COX-2, p22 phox and p47 phox, two NADPH oxidase subunits) [11] was investigated in isolated vessels. Arthritis score, radiographic score and plasma cytokine levels were also assessed. To decipher the mechanisms involved in the vascular effect of local cryotherapy, the number of leucocytes in the blood and plasma levels of osteoprotegerin (OPG), a regulator of bone metabolism able to induce endothelial activation/dysfunction [16, 17], were measured.

## Methods

### Animals and experimental groups

Six-week-old male Lewis rats were purchased from Janvier (Le Genest Saint Isle, France). Animals were kept under a 12h-12h light-dark cycle and allowed free access to food and water. The experimental procedures were approved by the local committee for ethics in animal experimentation n°2019-003-PT-5PR of Franche-Comte University (Besançon, France), and complied with the “Animal Research: Reporting In Vivo Experiments” ARRIVE guidelines. Experiments were conducted on separate series of rats used for the investigation of EA and plasma cytokines (“endothelial activation” series), leucocytes count in aorta and blood (“leucocyte infiltration” series) or endothelial function, and levels of plasma cytokines and OPG (“endothelial function” series).

### Induction and clinical evaluation of the arthritis model

Arthritis was induced by a single intradermal injection at the base of the tail of 120 μl of 1 mg of heat-killed *Mycobacterium butyricum* (Difco, Detroit, MI, USA) suspended in 0.1 ml of mineral oil (Freund's incomplete adjuvant (Difco, Detroit, MI, USA)). A control group received 120 μl of saline solution. The AIA model shows a rapid progression of a robust and easily measurable polyarthritis, characterized by severe erythema, diffuse soft tissue swelling with complete ankyloses and malformations in the paws, and reduced locomotor activity. The onset of clinical signs of arthritis was observed as early as day 10 post-immunization and all rats exhibited symptoms at day 14 post-immunization. The global arthritis score reached a maximum between days 20 and 24 and then decreased sharply until day 60 when it stabilized at a low level [18]. Rats were daily weighed and monitored for clinical signs of arthritis. The scoring system was employed as follows: arthritis of one finger scores 0.1, weak and moderate arthritis of one big joint (ankle or wrist) scores 0.5, and intense arthritis of one big joint scores 1. Tarsus and ankle were considered as the same joint. Sum of joints scores of 4 limbs leads to an arthritis score of a maximum of 6 per rat.

### Local cryotherapy protocol

Local cryotherapy was applied to both hind paws twice a day at a 8-h interval (at 9 A.M. and 5 P.M.) using a cold spray (Ice Spray, Ghiaccio Spray®, Artasana Group, Italy), sprayed from a distance of 25 cm on the hind paws of the rats (9 applications of 5 seconds alternating with 10 seconds of pause, on each of the paws), from the onset of arthritis (day 10 post-immunization) to the acute inflammatory phase (day 24 post-immunization) according to the protocol described by Guillot et al. (2017) [13]. With this protocol, skin temperature of the hind paws decreased from 28.4 ± 0.2°C to 14.1 ± 0.1°C at the end of the application.

### Tissue collection

The day after the last cryotherapy application, the rats were anesthetized (pentobarbital, 60 mg/kg, *ip*). Blood was then withdrawn from the abdominal artery and centrifuged to obtain plasma, divided into aliquots, and stored at − 80°C until analysis. Thoracic aortas were removed and either immediately used for vascular reactivity experiments (“endothelial function” series), or frozen in liquid nitrogen and stored at − 80°C until qPCR analysis (“endothelial activation” series). In rats from “endothelial function” series, the small intestine was also collected to dissect the mesenteric vascular beds rapidly for the study on endothelial function. In rats from “leucocyte infiltration” series, whole blood was collected from the abdominal aorta and 1 ml was transferred in a K2E microtainer (BD Biosciences, Le Pont-de-Claix, France), thoracic aorta was carefully removed, rinsed in cold saline solution, weighted and these samples were immediately processed for flow cytometry analysis.

### Radiological examination and radiographic score assignment

Hind paws were removed and stored in 4% formalin to assess joint damage by X-ray radiography with a BMA High-Resolution Digital X-Ray (40 mV, 10 mA; D3A Medical Systems). A score of 0 to 20 was assigned to each paw divided into 5 items including swelling, cartilage destruction, osteoporosis, bone erosion, and new bone formation, according to the modified rating scale of Ackerman et al. (1979) [19].

### qRT-PCR analysis

Expression at the mRNA level of markers of endothelial activation or dysfunction was measured in thoracic aortas. Aortas were mechanically crushed using a glass pestle, all kept on a layer of dry ice. Total RNA was extracted using the RNeasy Fibrous Tissue Mini Kit (Qiagen, Hilden, Germany). cDNAs were synthesized from 0.5 μg of total RNA, using the iScript cDNA Synthesis kit (Bio-Rad Laboratories, Hercules, CA, USA). Quantitative PCR was performed using the iQ SYBR Green Supermix kit (Bio-Rad) and the MyIQ device (Bio-Rad). The sequences of the primers used to study the expression of the mRNA of adhesion molecules (ICAM-1, VCAM-1), cytokines/chemokines (CXCL-1, MCP-1 (CCL-2), MIP-1α (CCL-3), TNF-α, IL-6), and enzymes (Arginase-2, COX-2, p22 phox, and p47 phox), as well as β-actin and GAPDH used as references are referenced below (Table 1). All the samples were deposited in duplicates. Two negative controls were used on each plate: a RNAse-free water and a no-RT sample. The thermocycler conditions were 3 min at 95°C to allow polymerase activation then 40 PCR cycles at 95°C for 15 s and at 60°C for 60 s. Normalized and averaged fluorescence ratios of targets were used to calculate the fold changes in samples from the different rat groups.

**Table 1:** Primer sequences used in qRT-PCR

Target	Forward primer (5′➔3′)	Reverse primer (5′➔3′)
**ICAM-1**	TGC-CTG-CAC-TTT-GCC-CTG-GT	ACA-GGC-CCG-GGG-ATC-ACA-AC
**VCAM-1**	TTG-TTC-AAG-AGA-AAC-CAT-TTA-GTG-T	TCA-TCC-TCA-ACA-CCC-ACA-GG
**CXCL-1**	CCA-GCC-ACA-CTC-CAA-CAG-AGC-A	GGC-GCC-CCT-GTG-GCT-TGG-
**MCP-1 (CCL-2)**	GTG-TGA-TTT-GGA-ATG-TGA-TG	AAG-TGT-TGA-ACC-AGG-ATT
**MIP-1α (CCL-3)**	AGA-ACA-TTC-CTG-CCA-CCT	AAG-TGA-AGA-GTC-CCT-GGA-T
**TNF-α**	CCA-ATC-TGT-GTC-CTT-CTA-A	TTC-TGA-GCA-TCG-TAG-TTG
**IL-6**	GAC-CAA-GAC-CAT-CCA-ACT	TAG-GTT-TGC-CGA-GTA-GAC
**COX-2**	TTT-GCC-TCT-TTC-AAT-GTG	TTA-ATG-TCA-TCT-AGT-CTG-GA
**P22 phox**	ACC-TGA-CCG-CTG-TGG-TGA-A	GTG-GAG-GAC-AGC-CCG-GA
**P47 phox**	TCC-TAT-CCC-TAC-CCT-TGT	GAG-TCT-GAG-TCC-ATT-CCA
**β-Actin**	TAT-CGG-CAA-TGA-GCG-GTT-GC	TGC-CTG-GGT-ACA-TGG-TGG-TG
**GAPDH**	GGG-CAT-CCT-GGG-CTA-CAC-TG	GAG-GTC-CAC-CAC-CCT-GTT-GC

### Flow cytometry

Thoracic aortas were cut and placed in a digestion solution containing 1 mg/mL of collagenase A (Sigma-Aldrich, Darmstadt, Germany, ref:10103578001), 1 mg/mL of collagenase B (Sigma-Aldrich, ref:11088807001), 100 μg/mL of type IV DNase (Sigma-Aldrich, ref:D5025-15KU) mixed in RPMI 1640 medium supplemented with 5% Fetal Bovine Serum (FBS) and incubated for 1h at 37°C. The suspension obtained was then filtered through a 70-μm MACS SmartStrainer (Miltenyi Biotec, Bergisch Gladbach, Germany), and then cells were pooled and suspended in 3 mL of RPMI/FBS 5%. Cells extracted from aortas were counted and then ~1 million cells per sample were prepared for flow cytometry with the following antibody mix: CD45 BV510 (clone OX-1), CD3 APC (clone 1FA), CD11b/c BV650 (clone OX-42), CD4 PE-Cy7 (clone OX-35), CD8 BV7711 (clone OX-8), PE granulocytes (RP-1 Antigen) (clone RP-1) (BD Biosciences, Le Pont-de-Claix, France). Blood analysis was performed with 30 μl of total blood per sample in Trucount tubes (BD Biosciences) using the same mix of antibodies. These Trucount tubes contain a defined number of microbeads to evaluate absolute blood cell counts in rodents [20]. Cell viability was assessed with eBioscience Fixable Viability Dye eFluor 780 (Invitrogen, Waltham, MA, USA). In order to evaluate the lymphocyte subpopulations producing IL-17A in the blood and aorta, an intracellular labeling of this cytokine after stimulation with phorbol myristate acetate (PMA) (1 μg/mL) and ionomycin (25 ng/mL) was performed associated with BD Biosciences GolgiPlug in order to inhibit the secretion of this cytokine outside the cells. After 4h of stimulation, a mix of surface antibodies is added into the cells to separate the different populations: leucocytes, lymphocytes, CD4^+^ T cells, CD8^+^ T cells (CD45 BV510, CD3 APC, CD4 PEcy7, CD8 BV7711 respectively). After washing the cells were permeabilized (Cytofix/Cytoperm, BD Biosciences) to allow the binding of the FITC-conjugated anti-IL-17A (BD Biosciences) monoclonal antibody (clone TC11-18H10). The gating method used for identifying the different leucocyte subpopulations is presented in Supplementary Fig. 1. All samples were assessed on the BD LSR Fortessa flow cytometer. Data were expressed as the number of labeled cells per mg of the thoracic aorta and the number of labeled cells per μL of blood.

### Vascular reactivity

Endothelial function was measured in isolated aortic rings and mesenteric arteries collected from the same animal. Thoracic aorta was excised, cleaned of connective tissue, and cut into rings of ~2 mm in length. Rings were suspended in Krebs solution (mmoles/L: NaCl 118, KCl 4.65, CaCl_2_ 2.5, KH_2_PO_4_ 1.18, NaHCO_3_ 24.9, MgSO_4_ 1.18, glucose 12, pH 7.4), maintained at 37°C and continuously aerated with 95% O_2_ −5% CO_2_, for isometric tension recording in organ chambers. Third-order mesenteric arteries (2-mm segments) were placed in Krebs solution, mounted in organ chambers and threaded on two 40 μm diameter stainless steel wires. To measure isometric force, the artery segments were connected into a Multi Myograph System (Model 610M v.2.2, DMT A/S, Denmark), and data were recorded using Chart^TM^ Ver.7 (ADInstruments, France). Mesenteric arteries were set to a normalized internal circumference L_1_ (0.9L_100_) in accordance to passive wall tension-internal circumference relationship under a passive transmural pressure of 100 mm Hg. After an initial equilibration period of 30 min, viability of vessels was tested from their vasoconstriction to high extracellular KCl (100 mmoles/L). The presence of functional endothelium was confirmed by more than 80% relaxation to the endothelium-dependent agonist acetylcholine (Ach, 10^−6^ moles/L) after pre-constriction with phenylephrine (PE, 3.10^−5^ moles/L). The completeness of endothelial denudation was confirmed by the absence of relaxation to Ach (10^-6^ moles/L). Arteries were again allowed to equilibrate 30 min before the start of the experiments. To determine whether local cryotherapy improved the endothelial function, endothelium-dependent relaxation to Ach of aortic rings (10^-11^-10^-4^ moles/L) and mesenteric arteries (10^−9^–10^−4^ moles/L) with intact endothelium and previously constricted with phenylephrine (PE, 10^−6^ moles/L) was compared between untreated and treated AIA. Of note, at day 24 post-immunization, Ach-induced relaxation in aortas from AIA was not found significantly different from controls (data not shown). Therefore, endothelial function in the aorta and mesenteric arteries were studied at a time at which ED was present and has been widely described [21], *i.e*. day 33 post-immunization. Accordingly, in rats from “endothelial function” series, cryotherapy was applied from day 10 to day 33.

### Plasma measurements

Plasma levels of TNF-α, IL-1β, and OPG were measured using Milliplex magnetic bead panel kits (PPX-03-MXZTENA, Thermo Fisher, Courtaboeuf Cedex, France for TNF-α, IL-1β, and RBN1MAG-31K, Millipore, Molsheim, France for OPG) and then analyzed with a MAGPIX system (Luminex Corporation, Houston, TX, USA). IL-6 and IL-17A levels were measured by ELISA (R6000B kit, Rat IL-6 Quantikine ELISA Kit, Biotechne R&D Systems, Lille, France, and BMS635 Invitrogen kit, Rat IL-17A ELISA kit, Thermo Fisher, Vienna, Austria). The limit of detection for TNF-α, IL-1β, OPG, IL-17A, and IL-6 levels were 2.9, 13, 1.3, 1, and 14 pg/ml, respectively.

### Data and statistical analysis

Values were expressed as means ± SEM. Data were analyzed using GraphPad Prism or Sigma plot softwares. Relaxant responses to Ach were expressed as the percentage of relaxation of the contractile response to phenylephrine 10^−6^ moles/L. Concentration-response curves to Ach were compared by 2-way analysis of variance (ANOVA) for repeated measures. Comparison between two values was assessed by unpaired Student *t* test or Mann-Whitney test when data were not normally distributed. The analysis of the relationship between two parameters was determined by linear regression analysis and Spearman’s correlation coefficient was calculated between these variables. *P*<0.05 was considered statistically significant.

## Results

### Local cryotherapy reduced disease severity

**Fig. 1:**
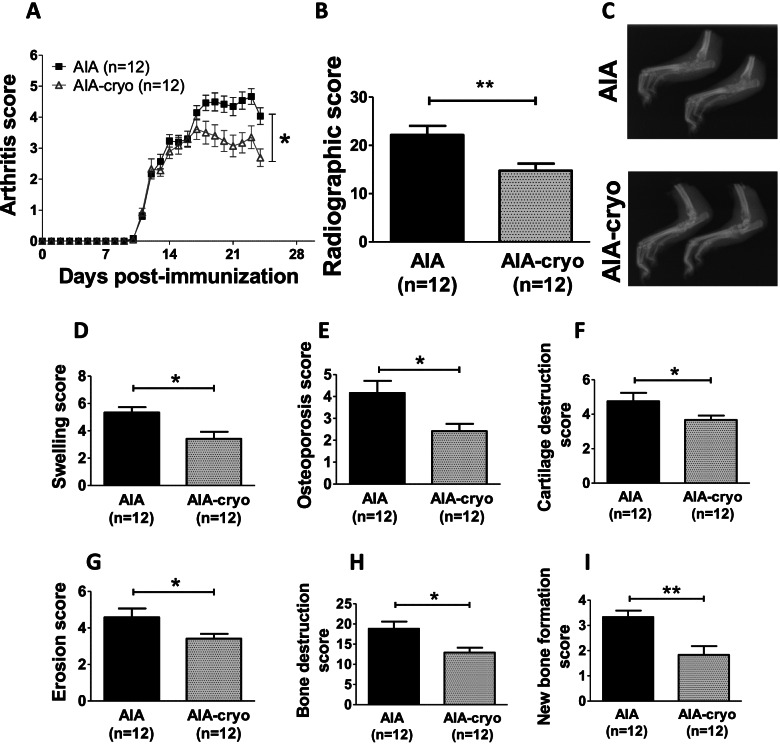
Effect of local cryotherapy on arthritis score and joint damage in AIA rats. Experiments were conducted in AIA rats from the “endothelial activation” series treated or not with local cryotherapy. **A** Time-course of arthritis score. **B** Radiographic score and **C** representative X-ray radiography of hind paws at the end of experiment. **D**–**I** Effect of cryotherapy on the different items of the radiographic score: swelling, cartilage destruction, osteoporosis, bone erosion and new bone formation. Results are expressed as means ± SEM (*n*=number of rats/group). *(*p*<0.05), **(*p*<0.01)

As compared to untreated AIA, local cryotherapy significantly reduced arthritis score (Fig. 1A, Table 2), the reduction reaching -41% in “endothelial activation” and “leucocyte infiltration” series, − 33% in “endothelial function” series at the end of the treatment. In addition, cryotherapy significantly reduced structural joint damage (Fig. 1B, C, Table 2). When scrutinizing the different elements of the radiographic score, it appeared that all the items (swelling, osteoporosis, cartilage destruction, bone erosion, new bone formation) were reduced by local cryotherapy (Fig. 1D–I).

**Table 2:** Clinical, structural, and biological parameters

	Arthritis score	Radiographic score	TNF-α (pg/ml)	IL-1β (pg/ml)	IL-6 (pg/ml)	IL-17A (pg/ml)	OPG (pg/ml)
**“Endothelial activation” series**							
Control	n.d.	n.d.	4.6 ± 0.9	26.1 ± 4.9	15.7 ± 1.8	2.7 ± 0.6	n.d.
AIA	4.0 ± 0.3	22.2 ± 1.9	15.3 ± 1.5^###^	64.5 ± 5.9^###^	65.0 ± 9.0^##^	25.8 ± 2.8^###^	n.d.
AIA-cryo	2.4 ± 0.3***	14.8 ± 1.5**	12.8 ± 1.2	59.7 ± 5.5	44.8 ± 3.1*	15.5 ± 2.1**	n.d.
**“Leucocyte infiltration” series**							
Control	n.d.	n.d.	n.d.	n.d.	n.d.	n.d.	n.d.
AIA	5.0 ± 0.2	21.0 ± 2.5	n.d.	n.d.	n.d.	n.d.	n.d.
AIA-cryo	3.0 ± 0.3**	14.7 ± 1.0*	n.d.	n.d.	n.d.	n.d.	n.d.
**“Endothelial function” series**							
Control	n.d.	n.d.	5.3 ± 4.5	17.2 ± 4.1	5.8 ± 2.4	5.5 ± 1.3	208.5 ± 26.7
AIA	4.6 ± 0.2	25.2 ± 1.9	5.1 ± 1.4	25.3 ± 3.7	28.3 ± 3.7^###^	23.6 ± 2.1^###^	220.8 ± 21.0
AIA-cryo	3.1 ± 0.2**	18.3 ± 1.7*	9.8 ± 5.1	31.3 ± 11.9	19.3 ± 3.4	18.1 ± 1.3*	170.3 ± 10.7*

Values are expressed as means ± SEM (*n*=7–12 rats per group). The treatment was applied from day 10 (onset of arthritis) to day 24 (acute inflammatory phase) for “endothelial activation” and “leucocyte infiltration” series, and until day 33 for “endothelial function” series. Cytokines and OPG were measured in plasma. n.d.: not determined

^##^ *p*<0.01, ^###^ *p*<0.001 *vs* control, * *p*<0.05, **p<0.01, *** *p*<0.001 *vs* AIA

### The AIA model was associated with endothelial activation that was blunted by cryotherapy

**Fig. 2:**
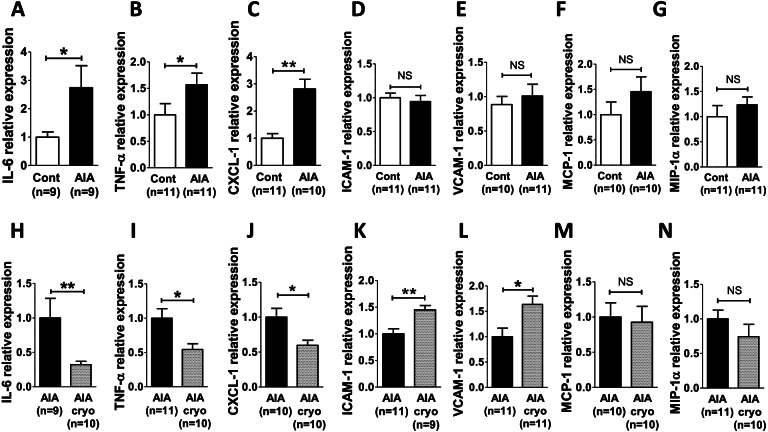
Expression of endothelial activation markers in AIA rats and effect of cryotherapy. **A**–**G** Effect of AIA on aortic mRNA expression of markers of endothelial activation as compared to control rats on day 24 post-immunization. **H**–**N** Effect of a daily treatment with local cryotherapy in AIA rats. Endothelial activation was assessed in thoracic aorta by measuring by qRT-PCR the mRNA expression of IL-6 (**A**, **H**), TNF-α (**B**, **I**), CXCL-1 (**C**, **J**), ICAM-1 (**D**, **K**), VCAM-1 (**E**, **L**), MCP-1 (CCL-2) (**F, M**) and MIP-1α (CCL-3) (**G**, **N**). Results are expressed as means ± SEM (*n*=number of rats/group). *(*p*<0.05), **(*p*<0.01). NS, non-significant

Since EA has never been explored in the AIA model, adhesion molecules, cytokines, and chemokines were first analyzed at mRNA levels in the aorta of AIA as compared to control rats. As shown in Fig. 2, at the acute inflammatory phase of arthritis, an increase in pro-inflammatory cytokines occurred in aortas as attested by the increased mRNA expression of IL-6 (Fig. 2A), TNF-α (Fig. 2B), and CXCL-1 (the IL-8 murine equivalent) (Fig. 2C) in AIA. By contrast, ICAM-1, VCAM-1, MCP-1 (CCL-2), and MIP-1α (CCL-3) mRNA expressions were unchanged (Fig. 2D–G). Second, the effect of cryotherapy on EA was assessed in AIA rats. As presented in Fig. 2, cryotherapy decreased aortic mRNA expression of IL-6 (−67%, *p*<0.01), TNF-α (−45%, *p*<0.05), and CXCL-1 (−40%, *p*<0.05) compared to untreated AIA (Fig. 2H–J). Regarding EA, while aortic mRNA expressions of VCAM-1 and ICAM-1 were increased by cryotherapy (Fig. 2K, L), no changes were observed with respect to mRNAs of MCP-1 (CCL-2) and MIP-1α (CCL-3) (Fig. 2M, N).

**Fig. 3:**
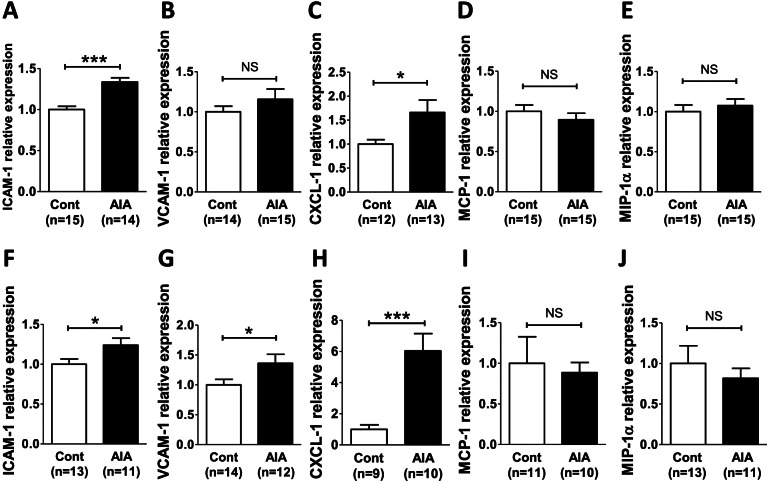
Aortic expression of endothelial activation markers at the preclinical stage and at onset of arthritis in AIA rats. mRNA expression of ICAM-1 (**A**, **F**), VCAM-1 (**B**, **G**), CXCL-1 (**C**, **H**), MCP-1 (CCL-2) (**D**, **I**), and MIP-1α (CCL-3) (**E**, **J**) was studied in thoracic aorta of control and AIA rats at the preclinical stage (day 4 post-immunization, **A**–**E**) and at the onset of arthritis (day 11 post-immunization, **F**–**J**) by qRT-PCR. Results are expressed as means ± SEM (*n*=number of rats/group). *(*p*<0.05), ***(*p*<0.001). NS, non-significant

Suspecting that induction of the mRNA expression of chemokines (CXCL-1, MCP-1 (CCL-2), MIP-1α (CCL-3)) and adhesion molecules (ICAM-1 and VCAM-1) occurred earlier in the arthritis course, their expression was studied in two additional groups of AIA and control rats at the preclinical phase (day 4 post-immunization) and at the onset of arthritis (day 11 post-immunization). As shown in Fig. 3, ICAM-1 and CXCL-1 expression increased in AIA rats at the preclinical phase (Fig. 3A, C) and at the onset of arthritis (Fig. 3F, H) while VCAM-1 increased only at the onset of arthritis (Fig. 3B, G). By contrast, the mRNA expression of MCP-1 (CCL-2) and MIP-1α (CCL-3) was unchanged whatever the phase of arthritis (Fig. 3D, E, I, J).

### AIA induced a robust leucocyte infiltration in the arterial wall that was dramatically reduced by cryotherapy

**Fig. 4:**
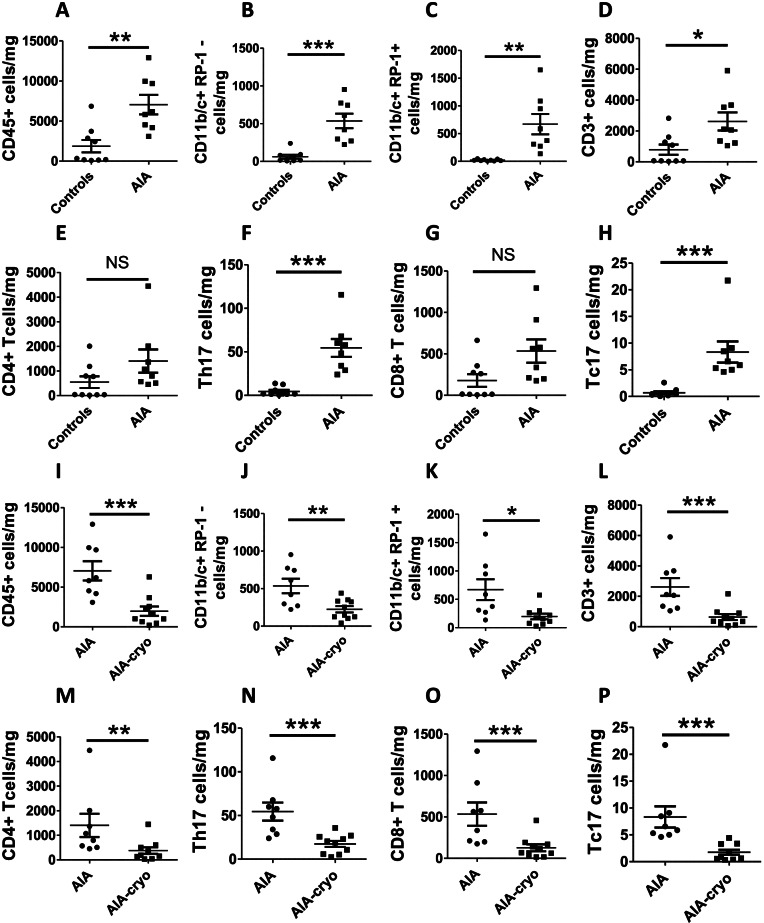
Leucocyte infiltration in aorta from AIA rats and effect of cryotherapy. **A**–**H** Flow cytometry analyses of CD45^+^ populations on rat thoracic aorta from control and AIA rats at the acute inflammatory phase (day 24 post-immunization). **I**–**P** Effect of local cryotherapy in AIA rats treated from day 11 to day 24 post-immunization. Different leucocyte subpopulations were studied: monocytes/macrophages CD11b/c^+^ RP-1^−^ (**B**, **J**), neutrophils CD11b/c^+^ RP-1^+^ (**C**, **K**), T lymphocytes CD3^+^ (**D**, **L**), CD4^+^ (**E**, **M**), CD8^+^ (**G**, **O**), CD4^+^ IL-17A^+^ (**F**, **N**) and CD8^+^ IL-17A^+^ (**H**, **P**) T cells. Gating strategies are shown in Supplementary Fig. 1A–J. Data are presented as the number of stained cells per mg of the aorta. Results are expressed as means ± SEM (n=10 rats/group). *(*p*<0.05), **(*p*<0.01), ***(*p*<0.001). NS, non-significant

To characterize vascular leucocyte infiltration in the AIA model, a flow cytometry analysis was conducted in aortas from AIA and control rats (Fig. 4). The results showed that AIA rats exhibited a strong leucocyte (CD45^+^ cells) infiltration as compared to controls (Fig. 4A). These leucocytes consisted in both innate immune cells, such as monocytes/macrophages and neutrophils (CD11b/c^+^ RP-1^–^ and CD11b/c^+^ RP-1^+^ cells, respectively) and in T lymphocytes (CD3^+^ cells) (Fig. 4B–D). While no significant increase was measured when looking at CD4^+^ and CD8^+^ T cells, likely because of a strong heterogeneity of the data, the increased number of IL-17^+^ CD4^+^ (Th17) and IL-17^+^ CD8^+^ (Tc17) T lymphocytes were observed in AIA as compared to controls (Fig. 4E–H).

As shown in Fig. 4I, subchronic treatment with cryotherapy induced a drastic decrease in aortic leucocyte infiltration, the effect being observed both on innate immune cells (Fig 4J, K) and T lymphocyte subpopulations (i.e., CD4^+^, CD8^+^, Th17, Tc17 cells) (Fig. 4L–P).

### Local cryotherapy improved endothelial function

**Fig. 5:**
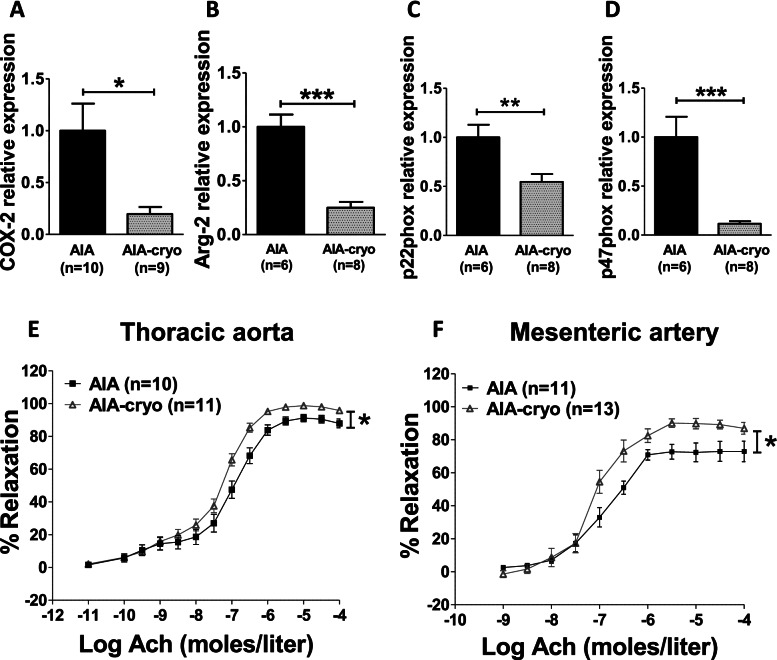
Effect of local cryotherapy on endothelial dysfunction in AIA rats. Experiments were conducted in AIA rats treated or not with local cryotherapy. Expression of endothelial pathways involved in AIA-associated endothelial dysfunction including COX-2 (**A**), Arg-2 (**B**), p22phox (**C**), and p47phox (**D**) was studied in aorta by qRT-PCR. Results are expressed as means ± SEM (*n*=number of rats/group). **E**, **F** Endothelial function was studied in rat on aortic rings and on mesenteric arteries by the measurement of acetylcholine (Ach)-induced vasorelaxation in endothelium-intact artery segments preconstricted with phenylephrine 10^−6^ moles/L. Results are expressed as means ± SEM (*n*=number of arterial rings, 6–7 rats/group). *(*p*<0.05), **(*p*<0.01), ***(*p*<0.001)

Previous studies demonstrated that upregulation of arginase-2, COX-2, and NADPH oxidase pathways was involved in endothelial dysfunction associated with AIA [11]. The present study showed that cryotherapy decreased aortic mRNA levels of these enzymes (Fig. 5A–D). Vascular reactivity experiments confirmed that these changes were associated with a reduction of the functional deterioration of endothelium. In isolated aortic rings, the vasodilating response to Ach was enhanced in cryotherapy-treated rats as compared to untreated rats (Fig. 5E). To confirm this result, endothelial function was evaluated in another vascular bed. As shown in Fig. 5F, local cryotherapy also enhanced Ach-induced vasorelaxation in mesenteric arteries.

### Local cryotherapy did not reduce the number of circulating leucocytes but decreased OPG and IL-17A plasma levels

**Fig. 6:**
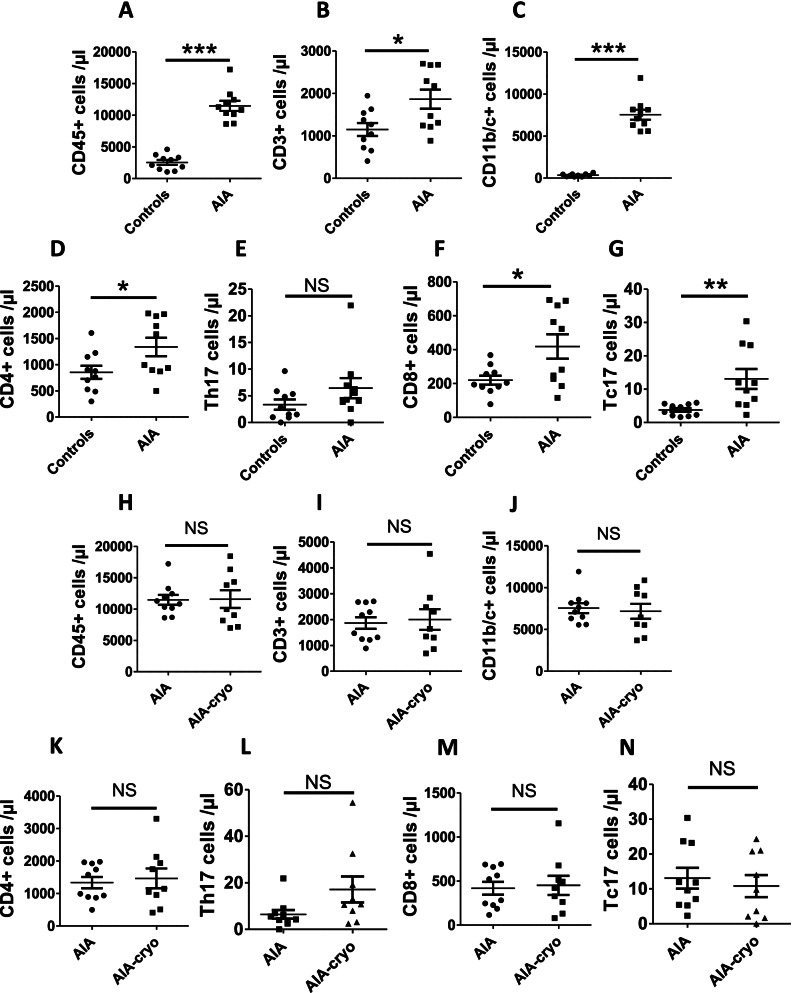
Circulating leucocytes in AIA rats and effect of cryotherapy. **A**–**G** Effect of AIA at the acute inflammatory phase (day 24 post-immunization) on leucocytes populations in the blood as compared to controls. **H**–**N** Effect of local cryotherapy on blood leucocytes and leucocyte subpopulations in AIA rats. Flow cytometry analysis assessed the absolute number of blood leucocytes (CD45^+^ cells, **A**, **H**) and of the different leucocyte subpopulations: CD3^+^ T lymphocytes (**B**, **I**), CD11b/c^+^ monocytes/macrophages (**C**, **J**), CD4^+^ (**D**, **K**), CD8^+^ (**F**, **M**) T cells, and intercellular IL-17A^+^ CD4^+^ (**E**, **L**) or CD8^+^ (**G**, **N**) T cells. Gating strategies are shown in Supplementary Fig. 1K-R. Data are expressed as number of stained cells per μl of blood. Results are expressed as means ± SEM (*n*=10 rats/group). *(*p*<0.05), **(*p*<0.01), ***(*p*<0.001). NS, non-significant

In the attempt to identify mechanisms linking local cryotherapy to systemic vascular effects, absolute number of circulating leucocytes and plasma levels of cytokines and OPG were measured. First, changes in circulating leucocytes were investigated in AIA rats as compared to controls. As shown in Fig. 6, AIA rats exhibited an increased number of leucocytes, T lymphocytes, and innate immune cells in the blood (Fig. 6A-C). Regarding T lymphocytes, as compared to controls, AIA rats showed a higher number of CD4^+^, CD8^+^ T, and Tc17 cells but the difference did not reach significance for Th17 cells (Fig. 6D–G). Local cryotherapy did not change the number of circulating leucocytes in AIA whatever the analyzed leucocyte subpopulation (Fig. 6H–N). Regarding cytokines and OPG, cryotherapy reduced plasma levels of OPG, IL-17A, and IL-6 but not IL-1β or TNF-α (Table 2).

### Correlations between endothelial activation/function, leucocyte infiltration, and markers of arthritis severity

**Fig. 7:**
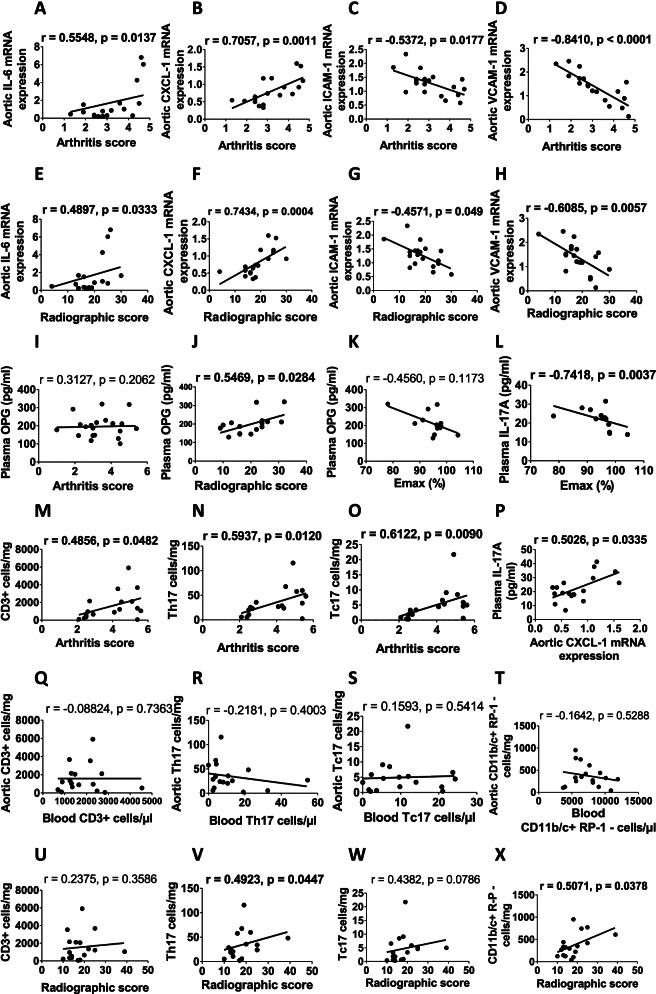
Correlations between clinical scores, plasma, and aortic cytokines expression, aortic adhesion molecule expression, aortic and blood count of leucocytes, and endothelial function in AIA rats. **A**–**X** Correlations were determined using Spearman correlation test in AIA rats treated or not with local cryotherapy. Endothelial function was expressed as the Emax of acetylcholine (Ach). Data in bold are significant (*p*<0.05)

Data on correlations between endothelial activation/function, leucocyte infiltration and markers of arthritis severity from treated and untreated AIA rats are presented in Fig. 7. Aortic vascular mRNA expressions of IL-6 and CXCL-1 were positively correlated while ICAM-1 and VCAM-1 negatively correlated with arthritis score and with radiographic score (Fig. 7A–H). Circulating levels of OPG did correlate neither with arthritis score nor with endothelial function (assessed by Emax of Ach) but correlated positively with radiographic score (Fig. 7I–K). By contrast, circulating IL-17A levels correlated negatively with endothelial function (assessed by Emax of Ach) and positively with aortic gene expression of CXCL-1 (Fig. 7L, P). Aortic number of T lymphocytes (CD3^+^), Th17 and Tc17 cells positively correlated with arthritis score (Fig. 7M–O) but not with the corresponding number of circulating T cells subsets (Fig. 7Q–S). Aortic monocytes/macrophages (CD11b/c^+^ RP-1^–^ cells) did not correlate with circulating monocytes/macrophages (Fig. 7T). Only the aortic number of monocytes/macrophages (CD11b/c^+^ RP-1^–^ cells) and Th17 cells positively correlated with radiographic score (Fig. 7U–X).

## Discussion

The new findings provided by this study are the following: (1) the AIA model was associated with an early aortic endothelial activation preceding endothelial dysfunction and (2) a subchronic treatment with local cryotherapy induced strong changes in the systemic vasculature including a reduction in endothelial activation, immune cell infiltration and endothelial dysfunction.

Cardiovascular diseases in RA are mainly the consequence of accelerated atherosclerosis [6, 7], which is secondary to endothelial cell dysfunction and activation [22]. These two endothelial changes are closely linked to each other, EA being able to induce ED but ED itself, notably by decreasing nitric oxide production, might be responsible for EA [23]. While ED can be easily measured by adapted methods in patients with RA [24], the evaluation of EA is challenging as it implies the measurement of circulating levels of adhesion molecules for which blood levels reflect, at least partially, their production by the inflamed joint. Animal models of arthritis confer the great advantage to collect vessels making them able to study the *vascular* expression of these markers. Surprisingly, whereas the rat AIA model reproduces most of the aspects of CV disease in RA including ED in large or small vessels and impaired cardiac function and structure [11, 25], EA has not been extensively studied in this model. The present study revealed that increased mRNA expression of adhesion molecules (ICAM-1) and CXCL-1 (IL-8) occurred as early as at the preclinical phase of AIA and reached its maximum at the onset of arthritis (ICAM-1, VCAM-1, IL-8) without any changes at the acute inflammatory phase. Aortic ED, attested by the altered relaxation response to endothelium-dependent vasorelaxant drugs was observed only at the acute inflammatory phase of this model (day 33), thus demonstrating that EA in large vessels is not the consequence of ED, but rather causal of ED in AIA. These data are in line with the study of Södergen et al. [26] in which patients with early RA presented high circulating levels of adhesion molecules whereas endothelial function measured by flow-mediated dilation was unaltered. EA is the *primum movens* for infiltration of immune cells within the arterial wall. Large artery inflammation measured by 18F-FDG-PET/CT was observed in arteries from early untreated RA patients and in Disease Modifying Antirheumatic Drugs (DMARDs)-treated patients with longstanding RA [27, 28]. Consistently, the AIA model exhibited a robust leucocyte infiltration at the acute inflammatory phase of the disease. The flow cytometry analysis identified immune cells including neutrophils, mononuclear cells, and T lymphocytes, i.e., cells classically associated with the atherosclerotic process (monocytes/macrophages, neutrophils [29, 30]), but also with systemic vascularitis (T cells, [31])). Of note, a persistent arterial inflammation was observed in RA subjects in long-term remission when treated with anti-TNF-α but not with other DMARDs [32], suggesting a differential effect of antirheumatic therapies on vascular inflammation.

Cryotherapy is widely used in RA as an adjunct therapy having the advantages to be cheap, safe, well-tolerated, and feasible at home. When subchronically applied on inflamed joints, cryotherapy by itself exerted positive effects on disease activity, as demonstrated in patients with RA [12, 33] and in AIA rats [13]. At the joint level, local cryotherapy decreased synovial Doppler hypersignal [34], IL-6, IL-17A, IL-1β but not TNF-α expression [13], and increased levels of metabolites of energy metabolism linked to antioxidant and anti-inflammatory activities [35]. The new finding of the present study is that local cryotherapy exerted positive effects remoted from the joint, improving all the aspects of AIA-induced systemic vascular pathology. Of note, a previous study in patients with ankylosing spondylitis showed that a cycle of 10 whole-body cryotherapy procedures with subsequent kinesiotherapy reduced plasma ICAM-1 levels as compared to kinesiotherapy alone [36], suggesting a positive effect on EA. Importantly, whether the effects of cryotherapy on the systemic vasculature would be maintained in the long-term after completion of the treatment was not investigated in the present study. Future studies using models of “chronic” arthritis (such as collagen-induced arthritis [37] or pristane-induced arthritis [38]) would be warranted to answer this point. In the present study, we showed that a *local* cryotherapy reduced the aortic gene expression of pro-inflammatory cytokines and vascular infiltration by immune cells. The expression of ICAM-1 and VCAM-1 mRNA were not decreased but increased by the treatment. A hypothesis is that this effect reflected the recruitment of immune cells necessary for inflammation resolution such as mononuclear cells. Indeed, ICAM-1 [39, 40] and VCAM-1 [41] are involved, either directly or through the increase in regulatory T cells after their endothelial transmigration, in macrophage efferocytosis. Efferocytosis is a specialized process that exerts not only the clearance of the apoptotic cells to prevent their secondary necrosis but also triggers several different anti-inflammatory and pro-resolving signaling pathways and thereby the termination of vascular inflammation [42]. An additional effect of cryotherapy was the reduction of ED, associated with the reduction of the overexpressed endothelial pathways [11] including arginase, COX, and NADPH oxidase.

In the light of these effects, the important point to address relates to the mechanisms linking cooling of hind paws to remote effects on the systemic vasculature. A first hypothesis (“immune hypothesis”) was that local cryotherapy reduced joint inflammation thereby reducing the release of multiple pro-inflammatory mediators leading to a reduction of the systemic immune activation. This hypothesis was discarded as the circulating leucocyte counts were not significantly reduced by the treatment. The second hypothesis (“endocrine hypothesis”) was that cryotherapy led to the reduction of circulating levels of mediators responsible for EA. Among them, IL-17A might be involved as this cytokine is known to induce endothelial activation and dysfunction [43] and was significantly reduced by local cryotherapy in AIA, both at the joint level [13] and in the blood ([13] and the present data). In favor of this hypothesis, circulating levels of IL-17A correlated with aortic gene expression of CXCL-1 and endothelial function in AIA rats. These results are congruent with a clinical study showing that plasma IL-17A levels correlated with ED in RA [44], and with a recent study in a cohort of 36 RA patients with a follow-up between 1970 and 2012 indicating that high levels of IL-17A were associated with both joint destruction and the occurrence of myocardial infarction [45]. Another culprit may be OPG, a regulator of bone turnover which was positively correlated with circulating markers of EA in patients with RA [46]. OPG is, at least in part, produced by the bone, and growing evidence indicated that OPG might be a link between bone metabolism and CV diseases, as it contributes to endothelial activation, dysfunction, and survival [47, 48]. In favor of this hypothesis, the present data showed that local cryotherapy reduced plasma levels of OPG. However, against this hypothesis, OPG levels did not correlate with endothelial function.

## Conclusion

In conclusion, the present study in the AIA model demonstrated that a subchronic treatment with local cryotherapy reduced endothelial activation and dysfunction, as well as arterial inflammation. These data encourage to study if cryotherapy protocols might result in additional benefits on vascular health in RA patients under anti-rheumatic drugs.

## Supplementary Information

**Additional file 1: Supplementary Figure 1**. Gating strategies used to analyze leucocyte subsets in aorta and blood samples. (A-F) A representative analysis of total leucocytes, granulocytes, monocyte/macrophages, total T cells, CD4^+^ and CD8^+^ T cells in aorta is shown. Cells were first identified based on their size and granularity (A, FSC *versus* SSC) and then total leucocytes were identified on the expression of the pan-leucocyte marker, CD45 (B). Then, viable CD45^+^ cells were selected using the Fixable Viability Dye eFluor 780 (FvD) (C). In these viable CD45^+^ cells, monocytes/macrophages and neutrophils were distinguished based on the expression of CD11b/c and granulocytes (RP-1 antigen) (E). T cells were identified in viable CD45^+^ cells by the expression of CD3 (D). In these viable CD3^+^ T cells, CD4^+^ and CD8^+^ T cells were distinguished based on the expression of CD4 and CD8, respectively (F). (G-J) A representative analysis of IL-17-producing T cells in aorta is shown. Cells were first identified based on their size and granularity (FSC *versus* SSC), their viability (FvD low) and the expression of CD45. Then, Th17 cells were identified by the expression of CD3 and CD4 (G) and intracellular expression of IL-17A (H), while Tc17 were identified by the expression of CD3 and CD8 (I) and intracellular expression of IL-17A (J). (K-R) A representative analysis of circulating total leucocytes, myeloid cells, total T cells, CD4^+^ and CD8^+^ T cells is shown. Cells were first identified based on their size and granularity (K, FSC *versus* SSC). This gate allows us to identify microbeads that were quantified in L. Total leucocytes were identified on the expression of the pan-leucocyte marker, CD45 (M). In these CD45^+^ cells, myeloid cells (monocytes/macrophages and neutrophils) were distinguished based on the expression of CD11b/c expression (O). T cells were identified in CD45^+^ cells by the expression of CD3 (N). In these CD3^+^ T cells, CD4^+^ and CD8^+^ T cells were distinguished based on the expression of CD4 and CD8, respectively (P). (Q-R) A representative analysis of circulating IL-17-producing T cells is shown. Cells were first identified based on their size and granularity (FSC *versus* SSC), their viability (FvD low) and the expression of CD45. In these viable CD3^+^ T cells, CD4^+^ and CD8^+^ T cells were distinguished based on the expression of CD4 and CD8, respectively. Then, Tc17 were identified by the expression of CD8 and intracellular expression of IL-17A (Q), while Th17 cells were identified by the expression of CD4 and intracellular expression of IL-17A (R).

## Data Availability

The datasets used and/or analyzed during the current study are available from the corresponding author on reasonable request.

## Abbreviations

Ach: Acetylcholine
AIA: Adjuvant-induced arthritis
APC: Allophycocyanin
BV: Brilliant violet
cDNA: Complementary DNA
CD: Cluster of differentiation
CCL: Chemokine (C-C motif) ligand
COX: Cyclooxygenase
CV: Cardiovascular
CXCL: Chemokine (C-X-C motif) ligand
DMARDs: Disease-modifying antirheumatic drugs
DNase: Deoxyribonuclease
EA: Endothelial activation
ED: Endothelial dysfunction
ELISA: Enzyme-linked immunosorbent assay
Emax: Maximal effect
FBS: Fetal bovine serum
FDG-PET/CT: Fluorodeoxyglucose-positron emission tomography with computer tomography
FITC: Fluorescein isothiocyanate
GAPDH: Glyceraldehyde-3-phosphate dehydrogenase
ICAM: Intercellular adhesion molecule
IL: Interleukin
MCP: Monocyte chemoattractant protein
MIP: Macrophage inflammatory protein
mRNA: Messenger RNA
NADPH: Nicotinamide adenine dinucleotide phosphate
OPG: Osteoprotegerin
PCR: Polymerase chain reaction
PE: Phycoerythrin
PEcy: Phycoerythrin-cyanine
PMA: Phorbol myristate acetate
RA: Rheumatoid arthritis
RNase: Ribonuclease
RPMI: Roswell Park Memorial Institute medium
RP-1: Monoclonal antibody against rat neutrophils
RT: Reverse transcription
SEM: Standard error of the mean
Tc: T cytotoxic
Th: T helper
TNF: Tumor necrosis factor
VCAM: Vascular cell adhesion molecule
