# Supplementary material for: Effects of local cryotherapy on systemic endothelial activation, dysfunction, and vascular inflammation in adjuvant-induced arthritis (AIA) rats
Source: Arthritis Res Ther. 2022 Apr 29;24:97. doi: 10.1186/s13075-022-02774-1 (PMC9052534; doi:10.1186/s13075-022-02774-1)
Supplement: Supplementary file 1 — Additional file 1: Supplementary Figure 1. Gating strategies used to analyze leucocyte subsets in aorta and blood samples. (A-F) A representative analysis of total leucocytes, granulocytes, monocyte/macrophages, total T cells, CD4+ and CD8+ T cells in aorta is shown. Cells were first identified based on their size and granularity (A, FSC versus SSC) and then total leucocytes were identified on the expression of the pan-leucocyte marker, CD45 (B). Then, viable CD45+ cells were selected using the Fixable Viability Dye eFluor 780 (FvD) (C). In these viable CD45+ cells, monocytes/macrophages and neutrophils were distinguished based on the expression of CD11b/c and granulocytes (RP-1 antigen) (E). T cells were identified in viable CD45+ cells by the expression of CD3 (D). In these viable CD3+ T cells, CD4+ and CD8+ T cells were distinguished based on the expression of CD4 and CD8, respectively (F). (G-J) A representative analysis of IL-17-producing T cells in aorta is shown. Cells were first identified based on their size and granularity (FSC versus SSC), their viability (FvD low) and the expression of CD45. Then, Th17 cells were identified by the expression of CD3 and CD4 (G) and intracellular expression of IL-17A (H), while Tc17 were identified by the expression of CD3 and CD8 (I) and intracellular expression of IL-17A (J). (K-R) A representative analysis of circulating total leucocytes, myeloid cells, total T cells, CD4+ and CD8+ T cells is shown. Cells were first identified based on their size and granularity (K, FSC versus SSC). This gate allows us to identify microbeads that were quantified in L. Total leucocytes were identified on the expression of the pan-leucocyte marker, CD45 (M). In these CD45+ cells, myeloid cells (monocytes/macrophages and neutrophils) were distinguished based on the expression of CD11b/c expression (O). T cells were identified in CD45+ cells by the expression of CD3 (N). In these CD3+ T cells, CD4+ and CD8+ T cells were distinguished based [file 13075_2022_2774_MOESM1_ESM.docx]

**Effects of local cryotherapy on systemic endothelial activation, dysfunction and**

**vascular inflammation in adjuvant-induced arthritis (AIA) rats**

C. Peyronnel^a^, P. Totoson^a^, V. Petitcolin^a^, F. Bonnefoy^b^, X. Guillot^c^, P. Saas^b^,

F. Verhoeven^a, d^, H. Martin^a,*^, C. Demougeot^a,*, #^

^a^ PEPITE EA4267, FHU INCREASE, Univ. Bourgogne Franche-Comté, F-25000 Besançon, France

^b^ INSERM UMR 1098 RIGHT, EFS BFC, Univ. Bourgogne Franche-Comté, LabEX LipSTIC, F-25000 Besançon, France

^c^ Service de Rhumatologie, CHU Felix Guyon, Saint-Denis, Ile de la Réunion, France

^d^ Service de Rhumatologie, CHRU Besançon, F-25000 Besançon, France

## ^#^ Corresponding author:

Prof. Céline DEMOUGEOT

EA 4267 UFR Sciences de Santé, 19 rue Ambroise Paré, bâtiment S, 25030 BESANCON cedex, FRANCE.

Tel: (33) 3 63 08 23 25

E-mail address: cdemouge@univ-fcomte.fr

^*^ These authors contributed equally to this work.

**Running title:** *Effect of local cryotherapy on systemic endothelial activation in arthritis*

**Supplementary Figure 1**

**
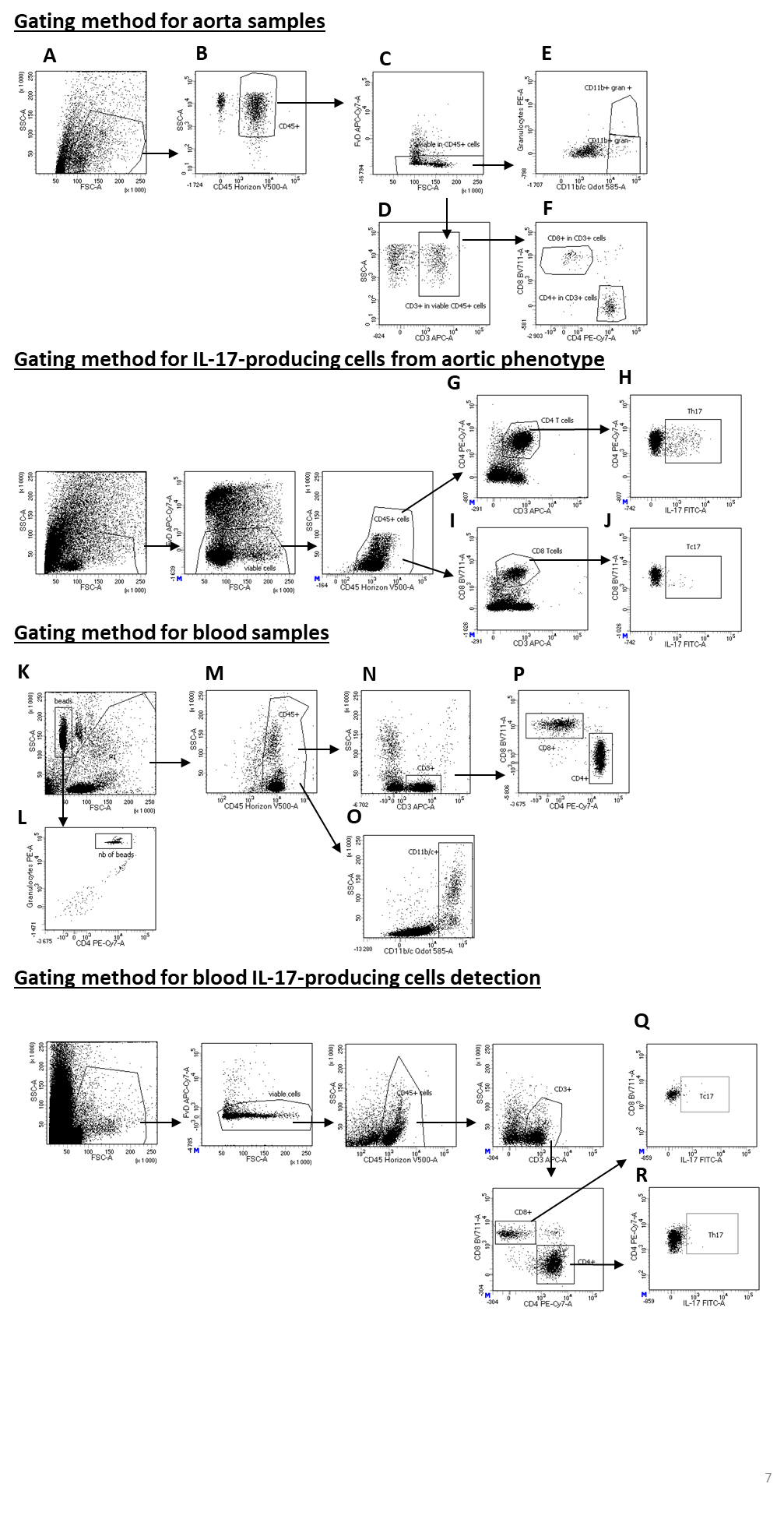
**

**Supplementary Figure 1. Gating strategies used to analyze leucocyte subsets in aorta and blood samples. (A-F)** A representative analysis of total leucocytes, granulocytes, monocyte/macrophages, total T cells, CD4^+^ and CD8^+^ T cells in aorta is shown. Cells were first identified based on their size and granularity (**A**, FSC *versus* SSC) and then total leucocytes were identified on the expression of the pan-leucocyte marker, CD45 (**B**). Then, viable CD45^+^ cells were selected using the Fixable Viability Dye eFluor 780 (FvD) (**C**). In these viable CD45^+^ cells, monocytes/macrophages and neutrophils were distinguished based on the expression of CD11b/c and granulocytes (RP-1 antigen) (**E**). T cells were identified in viable CD45^+^ cells by the expression of CD3 (**D**). In these viable CD3^+^ T cells, CD4^+^ and CD8^+^ T cells were distinguished based on the expression of CD4 and CD8, respectively (**F**). **(G-J)** A representative analysis of IL-17-producing T cells in aorta is shown. Cells were first identified based on their size and granularity (FSC *versus* SSC), their viability (FvD low) and the expression of CD45. Then, Th17 cells were identified by the expression of CD3 and CD4 (**G**) and intracellular expression of IL-17A (**H**), while Tc17 were identified by the expression of CD3 and CD8 (**I**) and intracellular expression of IL-17A (**J**). **(K-R)** A representative analysis of circulating total leucocytes, myeloid cells, total T cells, CD4^+^ and CD8^+^ T cells is shown. Cells were first identified based on their size and granularity (**K**, FSC *versus* SSC). This gate allows us to identify microbeads that were quantified in **L.** Total leucocytes were identified on the expression of the pan-leucocyte marker, CD45 (**M**). In these CD45^+^ cells, myeloid cells (monocytes/macrophages and neutrophils) were distinguished based on the expression of CD11b/c expression (**O**). T cells were identified in CD45^+^ cells by the expression of CD3 (**N**). In these CD3^+^ T cells, CD4^+^ and CD8^+^ T cells were distinguished based on the expression of CD4 and CD8, respectively (**P**). **(Q-R)** A representative analysis of circulating IL-17-producing T cells is shown. Cells were first identified based on their size and granularity (FSC *versus* SSC), their viability (FvD low) and the expression of CD45. In these viable CD3^+^ T cells, CD4^+^ and CD8^+^ T cells were distinguished based on the expression of CD4 and CD8, respectively. Then, Tc17 were identified by the expression of CD8 and intracellular expression of IL-17A (**Q**), while Th17 cells were identified by the expression of CD4 and intracellular expression of IL-17A (**R**).
